# Parsimonious Scenario for the Emergence of Viroid-Like Replicons De Novo

**DOI:** 10.3390/v11050425

**Published:** 2019-05-09

**Authors:** Pablo Catalán, Santiago F. Elena, José A. Cuesta, Susanna Manrubia

**Affiliations:** 1 Biosciences, College of Life and Environmental Sciences, University of Exeter, Exeter EX4 4QD, UK; pablocatalanfdez@gmail.com; 2 Grupo Interdisciplinar de Sistemas Complejos (GISC), Madrid, Spain; cuesta@math.uc3m.es; 3 Instituto de Biología Integrativa de Sistemas (I2SysBio), CSIC-Universitat de València, Paterna, 46980 València, Spain; santiago.elena@csic.es; 4 The Santa Fe Institute, Santa Fe, NM 87501, USA; 5 Departamento de Matemáticas, Universidad Carlos III de Madrid, 28911 Leganés, Spain; 6 Instituto de Biocomputación y Física de Sistemas Complejos (BiFi), Universidad de Zaragoza, 50018 Zaragoza, Spain; 7 Institute of Financial Big Data (IFiBiD), Universidad Carlos III de Madrid–Banco de Santander, 28903 Getafe, Spain; 8 National Biotechnology Centre (CSIC), 28049 Madrid, Spain

**Keywords:** viroid, RNA secondary structure, population dynamics, computational simulations, structure enumeration, simple replicators, modular evolution

## Abstract

Viroids are small, non-coding, circular RNA molecules that infect plants. Different hypotheses for their evolutionary origin have been put forward, such as an early emergence in a precellular RNA World or several de novo independent evolutionary origins in plants. Here, we discuss the plausibility of de novo emergence of viroid-like replicons by giving theoretical support to the likelihood of different steps along a parsimonious evolutionary pathway. While Avsunviroidae-like structures are relatively easy to obtain through evolution of a population of random RNA sequences of fixed length, rod-like structures typical of Pospiviroidae are difficult to fix. Using different quantitative approaches, we evaluated the likelihood that RNA sequences fold into a rod-like structure and bear specific sequence motifs facilitating interactions with other molecules, e.g., RNA polymerases, RNases, and ligases. By means of numerical simulations, we show that circular RNA replicons analogous to Pospiviroidae emerge if evolution is seeded with minimal circular RNAs that grow through the gradual addition of nucleotides. Further, these rod-like replicons often maintain their structure if independent functional modules are acquired that impose selective constraints. The evolutionary scenario we propose here is consistent with the structural and biochemical properties of viroids described to date.

## 1. Introduction

Since their discovery in 1971 [1], viroids have elicited both amazement and attention. Despite a small size of a few hundred nucleotides (nt) and a non-coding RNA sequence, these RNA molecules behave as competent and persistent replicators in plants, to our current knowledge, their only natural hosts. The origin of viroids has been one of the most controversial issues regarding these small pathogens. So far, hypotheses to explain the origin of viroids fall into three categories: first, those seeking for an origin from other extant cellular RNAs such as group I introns [2] or the snRNA U1 component of spliceosome [2]; second, it has been suggested that viroids originated from Ty-1-like retroelements or retroviruses by deletions of internal sequences [3]; finally, the third most popular hypothesis in the light of viroid physico-chemical and structural properties, suggests a possible ancestral origin of viroids in a precellular RNA World [4,5]. For a detailed review of the several hypotheses, see [6,7]. Nonetheless, all these hypotheses have been strongly criticized on the basis of spurious sequence similarities (see, e.g., [8] and E. V. Koonin’s comment in [9]).

The sequence and the structure of existing viroids must have evolved as a response to a variety of selection pressures and evolutionary constraints to guarantee their successful replication and propagation. Different structural domains of viroids, occasionally overlapping in sequence [10], are related to a variety of functions such as cell-to-cell and systemic movements, replication, circularization, or pathogenicity, and to ribozyme-like activities such as self-cleavage of multimeric intermediates of replication into individual genomes [7,10,11,12]. While compact secondary structures (especially the rod-like fold characteristic of Pospiviroidae) have been identified as a constraint for viroid evolution [13], their preservation seems essential to avoid degradation and inactivation [7] and to minimize the effect of deleterious mutations [14,15]. Still, their rod-like structures are less stiff than typical double-stranded RNA (dsRNA), a feature that may as well play a functional role, as it may facilitate recognition by RNA polymerases that transcribe dsRNA templates into single-stranded RNAs (ssRNA) [5,12].

Evidence for rapid evolution in viroids is mounting. As early as in 1996, Theodor O. Diener noted that “Results from site-directed mutagenesis experiments indicate that, upon exposure to selective pressures, viroids can evolve extremely rapidly, with another, fitter, component of the quasi-species often becoming dominant within days or weeks.” [6]. Viroids do behave as quasispecies [16], meaning that new mutations should be fixed through evolution and co-evolution with their molecular environment, a dynamical process that has been amply documented in viruses [17]. Though the small genomes of viroids allow faithful replication despite high mutation rates [18], the latter differ significantly in the two viroid families. The spontaneous mutation rates estimated for Avsunviroidae are between 1/400 for CChMVd [18] and 1/800 for ELVd [19], but that of PSTVd, and potentially other Pospiviroidae, is lower (in the range 1/7000–1/3800), being comparable to the mutation rate of RNA viruses [19]. In any case, such high mutation rates entail a population heterogeneity that has been confirmed in recent years. In a study with PLMVd where a peach tree was infected with a clonal inoculum, almost 4000 different sequences were identified after just six months of in-plant evolution [20]. Only about 50% of the positions were fully conserved, and sequences differing in approximately 50 mutations from the parental sequence (which was not recovered from the evolved population) were identified [20]. Significant variations in the consensus sequence of GYSVd have been described in relation to the life-history of the grapevines it infects [21], while sequence variability increased in HLVd as a result of sudden environmental changes [22]. Moreover, the amount and nature of variability in CEVd strongly depend on the citrus host variety infected and could revert from configurations when hosts are reverted [23]. The mounting number of observations speaking to the high heterogeneity of viroid populations, their small genomes, and their rapid sequence change strongly suggests that we might be in a difficult position to solve questions on viroid’s origin if evidence has to rely solely on sequence similarity. Actually, it was chiefly the lack of sequence homology [6] that led to the dismissal of hypotheses suggesting that viroids might have descended from a variety of cellular RNAs [7].

In some scenarios dealing with an old precellular origin of viroids, they are portrayed as RNA molecules with properties very similar to those of extant viroids [5,24], which happen to fit into the chemical conditions of an early RNA World. However and in a sense analogous to Spiegelman’s monster [25], viroids behave as minimal replicators. The in vitro evolution of the RNA genome of the Qβ phage, with an approximate length of 4500 nt, led to the eventual selection of a replicating RNA molecule (the monster) with just 218 nt [25]. This experiment demonstrated that, given the appropriate environment, complex genomes may reduce the set of functions they perform (or encode) to the minimum that guarantees their persistence. That scenario is akin to a top-down approach where viroids could have started as complex replicators in a (perhaps) simple molecular environment, bearing a larger array of functions that were subsequently lost to yield their present conformation [4,26]. A complementary conceptual scenario may also apply: in a bottom-up approach (or de novo origin), viroid-like replicators might have come into being as serendipitous replicating sequences that subsequently acquired additional functions from a surrounding complex molecular environment. This is the scenario that we aim to explore here.

Viroids are, indeed, just one class of members of a wider brotherhood of small non-coding infectious RNAs that may or may not express some autocatalytic activities. Other members of this brotherhood are the linear and circular RNA satellites (the latter also named as virusoids) that live as hyperparasites of other RNA viruses [27], other viroid-like RNAs such as the CarSV retroviroid integrated in the carnation genome [28], the cherry small-circular RNA (csc RNA1) [29], and the hepatitis δ virus (HDV), an infectious agent found only in humans co-infecting with hepatitis B virus (HBV) [30]. HDV is a circular RNA with a modular structure in which a region dubbed as the viroid-like domain has a hammerhead autocatalytic structure [31]. Phylogenetic studies provided support to the common origin of all these viroid-like structures [32], a finding that has been interpreted as supporting the hypothesis of their ancient precellular origin. The phylogenetic evidence is not incontestable [8], however, and null models that help distinguishing common origin from convergent evolution, a possibility difficult to dismiss given the small number of positions “conserved”, are urgently needed.

The main focus of this contribution is to examine quantitatively to what extent several of the known features of recognized viroids are easy to obtain through evolution. Many of their sequence and structural features can be obtained as a response to a number of selection pressures that are easy to cast in a simple fitness function: requiring an average folding energy (such that the folded structure is stable but able to be opened) may affect at once the G + C content and the fraction of paired bases; asking for structural robustness translates into favoring a high fraction of paired bases; specific motif sequences affect particular interactions with other molecules; certain structural motifs are responsible for ribozyme-like functions, such as the cleaving activity of hammerheads. Properties such as high structural robustness, here understood as the effect of point mutations in the secondary structure [14], might emerge if selection favors the formation of long helices in the structure. Our results lead us to hypothesize that viroid-like replicons might emerge de novo with relative ease through a parsimonious process triggered by small RNAs of indistinct origin. The requirement for the process to start is the appearance of a minimal combination of sequence and structural motifs for those RNAs to be recognized by the replication machinery of cells. Sequence elongation and acquisition of new functions might proceed step-wise without major difficulties.

## 2. Materials and Methods

### 2.1. Structural Properties of Extant Viroids

All the viroid species included in this study, as well as their accepted taxonomic classification (ICTV2018b release) are listed in Table 1. For each viroid species, the reference RNA genome sequences were downloaded from the NCBI Taxonomy Browser at https://www.ncbi.nlm.nih.gov/Taxonomy/Browser/wwwtax.cgi?id=12884 (last accessed 25 March 2019). Without loss of generality, we focused our study only on viroids from the two recognized families and purposely excluded other members of the viroid-like brotherhood. However, given the generality of our computational approach, our conclusions could be easily extrapolated to explain the origin and evolution of all these viroid-like organisms.

We folded the viroid circular RNA sequences into their minimum free energy (MFE) structure using the circfold routine from the ViennaRNA package Version 2.1.7 [33] and setting the temperature at 25 ${}^{\circ }$C. We then computed the number of hairpins and base pairs, the free energy of the structure, and the G + C content of the sequence (see Table 1).

### 2.2. Computational Model

We performed three sets of computational experiments. In the first one, we evolved populations of N=1000 circular RNA sequences of fixed length ℓ=300 nt using Wright–Fisher dynamics, letting them evolve for T= 20,000 generations. At each generation, sequences were folded into their MFE as described above. In order to select for fitter sequences, we used the fitness function:

(1) $$f=\exp\left(-\alpha E\left(1-\frac{E}{2E_{0}}\right)+\beta p+\gamma q\right),$$

where *E* is the free energy of the folded structure relative to the sequence length *ℓ*, $E_{0}$ is a reference energy value, *p* is the number of pairs in the structure, and *q* is the number of times a specific sequence motif is found in the sequence. In our simulations, we have taken $E_{0}=-0.433$ kcal mol${}^{-1}$ nt${}^{-1}$, which is representative of the folding energy per nucleotide of Pospiviroidae (e.g., HSVd in Table 1). As for the sequence motif, we have used CNGRRGRRAYCN as an example, which corresponds to the consensus sequence of the periodic motif in CCCVd-small and in PSTVd [24,34]. This fitness function penalizes those sequences whose free energy is far from $E_{0}\ell$ (when ℓ=300, this is equivalent to penalizing sequences far from −130.0 kcal mol${}^{-1}$). Functions similar to Equation (1) to select for more than one fitness trait have been used elsewhere [35]. We started all simulations with a randomly-sampled seed sequence. Sequences were chosen to reproduce in proportion to their fitness *f*, and mutations were chosen from a Poison distribution of parameter 0.1, which is equivalent to implementing a mutation rate per base pair $\mu_{P}=3.3\times 10^{-3}$.

In the second set of experiments, we started the evolutionary dynamics with sequences of length 30 nt. Dynamics were as in the first set of experiments, but an additional mutation mechanism was included: insertions occurred at a rate $\mu_{I}=3.3\times 10^{-3}$ per base, like point mutations. In either case, four different situations were analyzed: (a) only selecting for energy (α=2.0, β=0.0, γ=0.0); (b) selecting for energy and number of base pairs (α=2.0, β=2.0, γ=0.0); (c) selecting for energy and sequence motifs (α=2.0, β=0.0, γ=20.0); and (d) selecting for energy, pairs, and motifs (α=2.0, β=2.0, γ=20.0). Parameters were chosen such that the (1–3) selection pressures acting on the molecules were of similar strength.

In the third set of experiments, we evaluated the likelihood that rod-like molecules of length 130 nt that had evolved under the conditions specified in (d) of the second set of experiments would maintain their folded structure under recombination with a hammerhead ribozyme. For simplicity, the sequence 5${}^{\prime }$-GAAGAGUCUGUGCUAAGCACACUGACGAGUCUGUGAAAUGAGACGAAACUCUUU-3${}^{\prime }$, corresponding to the hammerhead ribozyme of avsunviroids [36] was added to different loops (terminal hairpin loops, internal loops, or bulges) of the rod-like structures. The complete sequence was subsequently folded into its minimum free energy structure to evaluate if both the paired structure of the evolved rod-like molecule and the hammerhead structure were maintained upon addition of the two modules.

## 3. Results

In order to quantify the ease with which viroid-like properties could emerge in evolutionary scenarios, we have addressed different aspects. First, we have calculated expected features of typical structures for short RNA sequences (of length up to 30 nt) and for sequences with the lengths of the viroids analyzed. Second, we have performed numerical simulations of circular RNA populations under a variety of assumptions and compared several properties of evolving populations with those of extant viroids.

### 3.1. Quantitative Properties of Viroid-Like RNA Sequences and Folds

#### 3.1.1. Structural Properties of Circular RNAs

The folding constraint of circular RNA sequences poses severe biases on the feasibility and abundance of the different structures. Counting how many structures a circular sequence of length *ℓ* can fold into, having *h* hairpins and *p* base pairs, is a nontrivial combinatorial problem that has nevertheless been solved with the help of generating functions [37]. If $v_{\ell ,p,h}$ denotes this number, then

(2) $$V\left(z,w,u\right)=\sum_{\ell =0}^{\infty }\sum_{p=0}^{\infty }\sum_{h=0}^{\infty }v_{\ell ,p,h}z^{\ell }w^{p}u^{h}$$

defines its generating function, which turns out to be

(3) $$\begin{array}{cc} V(z,w,u)= & \;\frac{1}{2z^{2s}w^{s}}\left[B\left(z^{2},w^{2},u^{2}\right)\left(1+z^{2}w-z^{2s}w^{s}\right)-B{\left(z,w,u\right)}^{2}\left(1-z^{2}w+z^{2s}w^{s}\right)\right]\\ & -\sum_{k=1}^{\infty }\frac{\varphi (k)}{k}\log\left[1-z-B\left(z^{k},w^{k},u^{k}\right)\right]-B\left(z,w,u\right)\frac{1-uz^{m}}{1-z}, \end{array}$$

where φ(k) is Euler’s totient function, B(z,w,u) solves the equation

(4) $$B\left(z,w,u\right)=\frac{z^{2s}w^{s}}{1-z^{2}w}\left(\frac{1}{1-z-B(z,w,u)}-B\left(z,w,u\right)-\frac{1-uz^{m}}{1-z}\right),$$

and *m* and *s* are the minimum number of base pairs in a stem and of unpaired bases in a hairpin loop, respectively, two energetic constraints on folding (see [37] for full details).

Complicated as this may look, it is not difficult to extract asymptotic expressions for $v_{\ell ,p,h}$ when the sequences are long [37]. The total number of structures grows as

(5) $$v_{n}=\sum_{p=0}^{\infty }\sum_{h=0}^{\infty }v_{\ell ,p,h}∼1.45811\ell^{-5/2}{\left(1.84892\right)}^{\ell },$$

a result analogous to that obtained for the number of structures for open RNA chains [38]. On the other hand, the probability $v_{\ell ,p,h}/v_{r}$ that a circular structure of length *ℓ* has *p* base pairs and *h* hairpins follows a bivariate normal distribution. In particular, the expected number of base pairs and hairpins grows with *ℓ* as

(6) $$E\left(p\right)∼0.286472\ell +0.773395+o\left(\ell^{-1}\right),\;E\left(h\right)∼0.0378631\ell +0.681247+o\left(\ell^{-1}\right).$$

Table 1 lists these expected values for the lengths of extant viroids.

We can further use Equation (3) to extract the exact count for short sequences using some symbolic algebra package. The results are listed in Table 2 for sequences shorter than 30 bases. It is worth stressing the dominance of rod-like structures for these short sequences. In particular, a third hairpin did not appear until length 21 nt, and even at length 29 nt, the abundance of rods over other structures was nearly seven-fold.

#### 3.1.2. Phenotype Sizes

The abundance of sequences yielding structures with specific properties cannot be directly estimated from Table 2, since the size of a phenotype (meaning in this case the total number of sequences folding into a specific shape) depends on its structural properties [39]. For example, the higher the number of pairs in an RNA secondary structure, the fewer the number of sequences compatible with such a structure [40]. Furthermore, structures with a low number of pairs are energetically disfavored [41], since stabilizing a structure with short stacks requires pairs of low free energy such as G-C, and this condition limits the number of sequences compatible with such structures. On the other hand, paired nucleotides have lower neutrality than unpaired ones (they admit fewer changes without modifying their paired condition) [42,43], so structures with many paired positions tend to have smaller phenotypic sizes. Eventually, it turns out that typical structures (the most abundant ones in Table 2) also have the largest phenotypes, so that the frequency of sequences folding into typical structures is much higher than the frequency of typical structures themselves. While the latter quantity can be derived from Table 2, the former requires full consideration of the sequence-to-MFE secondary structure map.

Our analytical results indicate that viroid-like folds, and especially rod-like shapes (those with h=2), become increasingly rare in the space of structures as sequence length grows. Equation (6) shows that the number of hairpins increases approximately one unit every 26 nt in the sequence. Still, the huge degeneracy between RNA sequence and structure predicts an astronomically large number of sequences folding into a vast majority of structures (including those of viroids). Results in [44] based on the computational exploration of the sequence-to-structure map in RNA [45] allows us to estimate the size *S* of any RNA structure with 2p paired nucleotides and u=ℓ−2p unpaired nucleotides as:

(7) $$S=v_{p}^{2p}v_{u}^{u},$$

where $v_{p}=1.17\pm 0.08$ and $v_{u}=2.79\pm 0.08$ are numerically-obtained quantities [44]. The number of sequences compatible with viroid structures varies between about $10^{46}$ for CCCVd and $10^{72}$ for CChMVd. However, note that a typical structure of length 246 nt harbors about 142 paired nucleotides, so its phenotype size become of order $10^{56}$. That size rises to about $10^{91}$ for a typical structure of length 399 nt (see Table 1 for a comparison between the number of pairs in each viroid and the number expected in random sequences of the same length).

#### 3.1.3. Probability of Specific Sequence Motifs in Random Sequences

It is well known that specific sequences in the viroid genome are related to functions that are essential to complete the viroid cycle. Such sequences have been identified in PSTVd to promote entry into the nucleus [46], in HSVd related to pathogenicity [47], in the central conserved region (CCR) of different *Pospiviroid* species to guarantee optimal replication [48], or in the hammerhead motif of Avsunviroidae for effective autocatalytic activity [49]. Indeed, viroid sequences are subjected to a diversity of selective pressures that lead to conserved positions and regions [12].

In general, the likelihood of the appearance of a specific sequence motif can be estimated as follows. As an illustration, consider CNGRRGRRAYCN, the consensus sequence of the periodic motif in CCCVd-small and in PSTVd [24,34], which will be later used in the numerical simulations. The calculation proceeds in the same fashion for any other case. This sequence has 5 fixed positions, 2 positions that can be occupied by any nucleotide, 4 positions by a purine, and 1 by a pyrimidine. There are $N=4^{2}\times 2^{4}\times 2^{1}=2^{9}$ possible sequences out of $4^{12}=2^{24}$ different ones. The likelihood that the motif CNGRRGRRAYCN appeares at a fixed position in a sequence (that is, assuming that there is a unique possible site for the initial C) was $2^{-15}\approx 3\times 10^{-5}$; in other words, the motif appears on average in three out of 100,000 molecules. However, if the initial position does not matter, this number significantly grows with the length of the molecule. Note that the probability that the motif appears at least once anywhere in a sequence of length *ℓ* is $1-{\left(1-2^{-15}\right)}^{\ell }\approx 2^{-15}\ell$. For random sequences of length ℓ=300, the motif will be found in almost 1% of the sequences.

The random appearance of a circular RNA sequence folding into a rod-like structure with a sequence motif that promotes interaction with other molecules (e.g., polymerases, RNases, or ligases) is therefore not just a possible event, but a highly likely one. The stochasticity inherent to this random matching between two dissimilar molecules might perhaps explain why Pospiviroidae use DNA-dependent RNA polymerase II instead of a nuclear RNA-directed RNA polymerase [7] or why PSTVd uses DNA ligase 1 to circularize the genomic RNA monomers [50]. Actually, the results above suggest that, in view of the ubiquity of circular RNAs in the cell, potential viroid-like replicons could be steadily generated, in the absence of any specific selection pressure, with high likelihood.

### 3.2. Evolutionary Dynamics of Circular RNAs of Fixed Length

An in silico evolutionary experiment with populations of circular RNAs of size 300 nt has been performed with the aim of addressing two main questions: Which secondary structures dominate in such populations when different selection pressures are applied? How likely is it to evolve from *Pospiviroid*-like structures to *Avsunviroid*-like structures (or the other way round)?

In all numerical experiments performed, an average energy of the folded structure similar to that observed in natural viroids (of similar length) was preferentially selected. This selection pressure responded to the observation of viroids maintaining a sufficiently low energy so as to fold into stable structures, but sufficiently high so as to be opened with relative ease for replication. A second selection pressure applied aimed at maximizing the number of pairs with the goal of favoring structural robustness. If this pressure was applied in the absence of selection for an average energy, the G + C content increased without restrictions, leading to highly-stable and robust structures that, however, did not show any plasticity and, as a consequence, would be very difficult to replicate. A third selection pressure regarded a specific sequence motif, where we chose CNGRRGRRAYCN as an example. For simplicity, let us represent by *P*(*M*) the situation where increasing the number of pairs (minimizing the distance to the sequence motif) increases fitness, while selection for an average energy *E* occurs in all cases.

**Figure 1:**
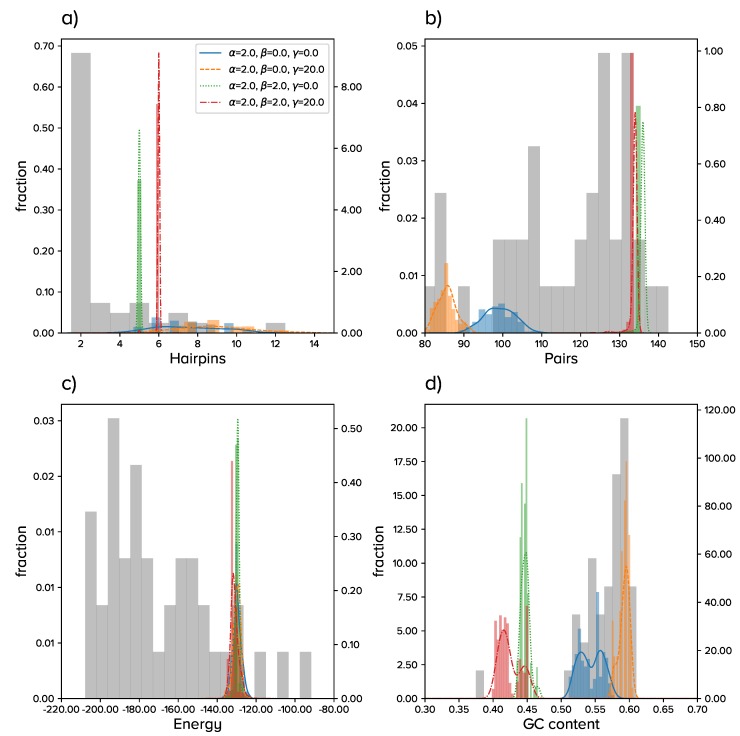
Summary of the results for the evolution of populations of circular RNA sequences of fixed length. Histograms in color correspond to numerical simulations with parameters specified in the legend. Lines are kernel density estimates of the underlying distribution. Grey bars correspond to viroids in Table 1. (**a**) Number of hairpins *h*; (**b**) number of base pairs *p*; (**c**) minimum free energy of the secondary structure; (**d**) G + C content.

Figure 1 summarizes the results of the four situations once the populations have reached a statistically-stable equilibrium. Colored curves correspond to numerical simulations (*E*, blue curve; E+M, orange curve; E+P, green curve; E+P+M, red curve), while grey histograms correspond to extant viroids and are displayed for comparison. The four situations can be grouped into two major qualitative behaviors: *E* resembles E+M, and E+P is similar to E+P+M.

If only energy or energy and a sequence motif were positively selected, we obtained populations highly heterogeneous in structure, with a broad distribution for the number of hairpins (from 4–12, with typical numbers around 6–9). Furthermore, the number of paired nucleotides followed broad distributions, with averages that kept relatively low as compared to most viroids. In order to maintain the average energy required in the simulations and as a result of the latter, the G + C content attained high values.

The high diversity of the former populations severely decreased when selection favoring the increase in the number of pairs was considered. This selective pressure led to populations with a lower number of hairpins (with the average around five or six), a significantly larger number of pairs, and in agreement, a lower G + C content. Note that a sequence of length *ℓ* with *h* hairpins (whose minimum size is three) has a maximum number of pairs $p_{\mathrm{mx}}=\left(\ell -3h\right)/2$. For ℓ=300 and h=5, $p_{\mathrm{mx}}=142$, so the number of pairs was close to maximal in these simulations. Our results show a relationship among number of pairs, folding energy, and G + C content. If $E_{0}$ decreased and since the number of pairs was already close to its highest possible values, the G + C content had to increase to meet the requirement. This would lead to sequences more similar to actual viroids, but even less plastic than those found in our simulations and holding a very low structural diversity in their populations. If $E_{0}$ decreased too much, the opening of those structures to perform the necessary functions via interaction with cellular components would be compromised.

Finally, we monitored the number of appearances of the sequence motif CNGRRGRRAYCN in all four situations. When the motif was selected for, it appeared almost immediately in the situation E+M, while it took a few thousand generations to emerge under E+P+M. The number of repetitions per sequence grew and reached over 20 appearances per sequence in E+M and about three appearances per sequence in E+P+M by the end of our simulations, though this number did not seem to have saturated. These results agree with the huge degeneracy of the sequence-to-structure map, which permits, to a good extent, the simultaneous and successful selection for structure and specific sequence motifs.

**Figure 2:**
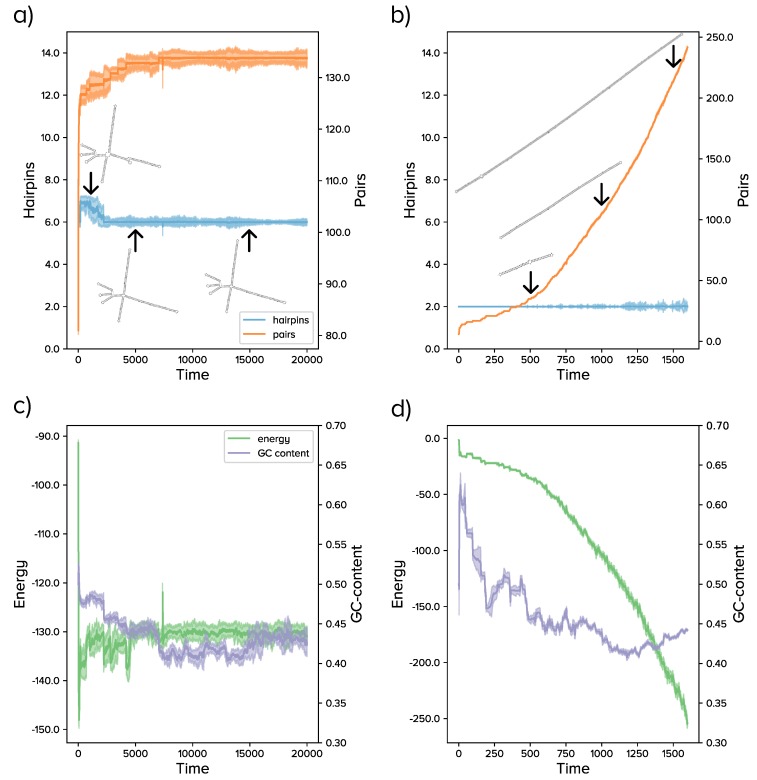
Evolutionary dynamics of RNA populations with fixed (**a**,**c**) and variable (**b**,**d**) length. In the former case, simulations start with a random sequences of length 300 nt; in the latter, of length 30 nt. (**a**) The number of hairpins decreased initially, but rapidly froze around six; the number of base pairs increased until a value near the maximum possible for that number of hairpins. The most abundant structure is shown at three different time points, as indicated by arrows. (**b**) As in (**a**), for sequences growing in length. (**c**) Dynamics of energy and G + C content for the same run shown in (**a**). (**d**) As in (**c**), for the run shown in (**b**).

The numerical results reported in this section suggest that selection of rod-like shapes was unlikely if evolution started with relatively long sequences. Requiring a high number of pairs without limiting the number of hairpins yielded long helices, but branched structures, with no less than five hairpins and a low population diversity. An example of the evolutionary dynamics in the situation E+P+M is represented in Figure 2a,c. Moreover, if the number of base pairs was positively selected and independent of other selective pressures, long stacks were locally fixed, since a large number of paired bases was however compatible with highly-branched structures. The effect of point mutations on structures with long helices was typically small, such that populations were trapped in structure space and minor changes in sequence found it hard to modify the overall structure [14,51], hence the number of hairpins. Our results support previous suggestions that viroids might be evolutionarily trapped due to adaptive constraints [13]. Here, we show that maximizing the number of pairs was indeed a constraint limiting innovation if only minor changes in sequence occurred. As a side result, it seems unlikely that the two viroid families were phylogenetically linked at a late evolutionary stage, that is for sequence lengths comparable to those of extant viroids. In the same vein, the emergence of viroid-like parasites through evolution of the large circular RNAs expressed in eukaryotes might be difficult [52,53].

### 3.3. Evolutionary Dynamics of Circular RNAs of Increasing Length

The possibility that extant viroids have evolved from large circular RNAs, either randomly or selected for other functions, seems unlikely. Trapping in configuration space might play a two-fold important role: evolution of an RNA molecule by point mutations might involve too large evolutionary times, while at the same time, it serves to preserve the original function. In particular, and as we have just shown, the structural plasticity of viroid-like structures, with many base pairs, was lower than average (compared to random sequences of the same length). Perhaps a more plausible evolutionary pathway for the emergence of viroid-like replicons would start with random short sequences whose length might increase concomitantly with functional selection. Among others, micro-RNAs (miRNA) [54] could be potential candidates to trigger the process that we describe in this section. Intriguingly, miRNAs and viroids have been found to share important structural features [55], among which the pervasive presence of stem- or rod-like secondary structures, respectively.

Numerical and analytical calculations demonstrated that hairpins [37,41,56] and small rods ([37] and this work) were highly preferred structures for short RNAs, so that these shapes were dominant in the absence of specific selection pressures. Circularization of short ssRNAs should occur frequently, among others, as a spontaneous product of the ligase activity exhibited by hairpin structures [57,58]. Notably, a ribozyme activity of hairpin-like structures was first described in plant virus satellite RNAs [59,60]. Indeed, it was later shown that the hairpin ribozyme of the satellite RNA of tobacco ringspot virus shows self-ligation activity in the presence of magnesium ions and low temperature [61].

We started the simulations in this section by taking as initial condition a population of circular RNAs of length 30 nt. They overwhelmingly folded into rod-like structures. The selection pressures applied were as in the former section. We analyzed two evolutionary mechanisms: addition of nucleotides and recombination with an independent hammerhead ribozyme. The addition of simple sequence repeats was an additional plausible mechanism for elongation, consistent with observations [62], that we did not consider explicitly here. The main question addressed was whether the rod-like structure that seeded the process was maintained along evolution.

#### 3.3.1. Growth through Insertion of Single Nucleotides

Simulations for growing sequences have been repeated in the four situations described, *E*, E+M, E+P, and E+P+M. As above, qualitative results grouped into two different pairs: *E* was akin to E+M, while more interesting results were obtained for E+P or E+P+M. If selection for the number of pairs was absent, sequences elongated at a slow pace initially, apparently more as a result of drift than as a consequence of any of the selective pressures acting on them. After evolution for several tenths of generations, sequence lengths reached sizes that varied between 300 nt and 500 nt, a growth that was accompanied by a significant increase in the number of hairpins. The number of base pairs was well below its maximum possible value, meaning that loops were frequent and/or large: populations were mainly formed by highly-unstructured molecules of low structural robustness. As expected, sequence motifs were absent in the *E* situation (they occasionally appeared, but were not fixed) and reached 15 repetitions (and growing) after about $6\times 10^{5}$ generations in the E+M situation.

The results of simulations for the situation E+P+M (qualitatively equivalent to E+P) are summarized in Figure 2b,d and compared with our numerical results in populations of sequences of constant length. At odds with what we observed in the latter case, the gradual addition of nucleotides preserved the initial number of hairpins and therefore yielded increasingly large rods. Examples of the most abundant structures at different time points are shown in Figure 2b, as indicated by arrows. The G + C content of the sequences stabilized at around 0.4–0.45 (compare with the red curve in Figure 1d), while the minimum folding energy decreased proportionally to the number of pairs. In just $10^{3}$ generations, the populations were ensembles of rod-like structures with sizes comparable to that of extant viroids and at least one copy of the sequence motif under selection.

#### 3.3.2. Growth through Modular Recombination

Some authors have described the viroid structure as a “collection of structural motifs which play specific functional roles in viroid replication, processing, transport, and pathogenesis” [12]. Though not explicitly discussed, this modular structure hints at the possibility that the different functional abilities of viroids could have been acquired through recombination with functional RNAs of different origins. Viroids formed through recombination of fragments present in other viroids have been described [63,64]. A highly-plausible case of modular recombination is provided by HDV [30], the unique animal pathogen described to date with a viroid-like non-coding domain [32] and a second domain coding for an antigen of independent evolutionary origin [65]. The possibility that independent RNA modules, functional in other molecular contexts, could have endowed bona fide viroids with new functions remains however as a hypothesis. Still, modular evolution has several advantages (as compared to direct evolution of longer molecules) [66], which may have been determinant in the early emergence of chemical function [56]. In this sense, the widespread occurrence of the hammerhead ribozyme in many prokaryotic and eukaryotic transcriptomes [67] and, more importantly, the observation that these hammerhead ribozymes in plant genomes are part of small circular RNAs related to Ty-3 LTR retrotransposons [68], which have been related with the origin of viral satellites and *Avsunviroid* [69], creates a molecular ecology in which the hammerhead structural motif would be abundant, hence increasing the likelihood to be acquired by proto-viroid sequences at early stages of their evolution.

**Figure 3:**
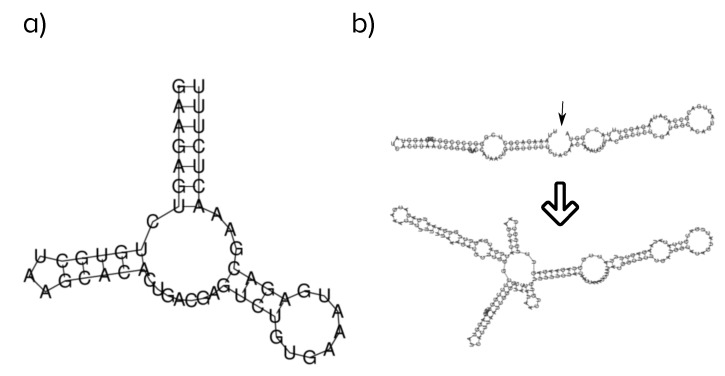

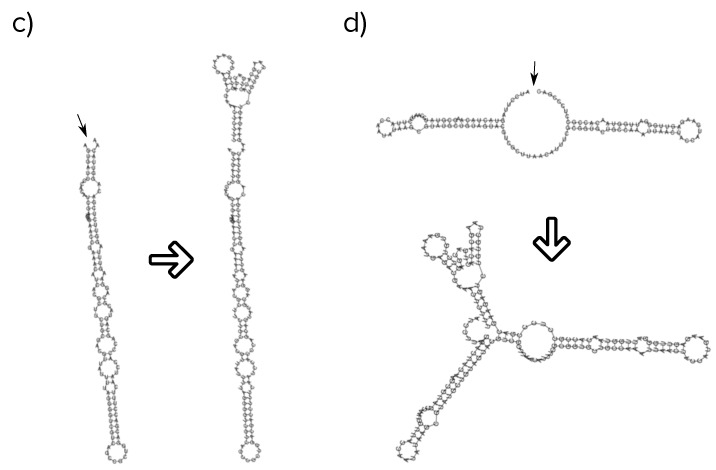
Effects of modular evolution in the structure of rod-like folds. Small arrows signal the recombination site; large arrows indicate the transformation of the structure upon recombination with the hammerhead. (**a**) Structure of the example hammerhead to be recombined with evolved sequences. (**b**) Part of the structure is maintained and part disrupted. The hammerhead structure is lost. (**c**) Both recombining structures are preserved. (**d**) The hammerhead structure is preserved, but the rod-like fold is partly disrupted.

As a representative example, we quantify here the likelihood that important functions of viroids dependent on their secondary structure would be preserved under modular evolution. First, we evolved single sequences as in the previous section until they reached length 130 nt. In all cases, they had a rod-like secondary structure, but there were variations, albeit narrow, in their energy, G + C content, or number of base pairs. At that point, we studied the effect of ligation of the evolved sequence to the hammerhead ribozyme of viroids [36] (see Materials and Methods). Figure 3 portrays the structure of the hammerhead and summarizes the possible outcomes of the process. The hammerhead structure was preserved in 12% of recombination events (two possible cases are illustrated in Figure 3c,d). Still, the two structures were relatively easy to preserve, for instance if recombination occurred at one of the terminal hairpins. Specifically, we found that this happened in 8% of cases; see Figure 3c.

It is likely that the very rod-like structure plays a role towards increasing its preservation under recombination, in the same sense that such structures are more robust to mutations [14,51]. In a different context, the modular combination of two RNA structures resulted in 4% of ligation events preserving the structures of the independent modules, which had a lower fraction of paired bases [66].

## 4. Discussion

**Figure 4:**
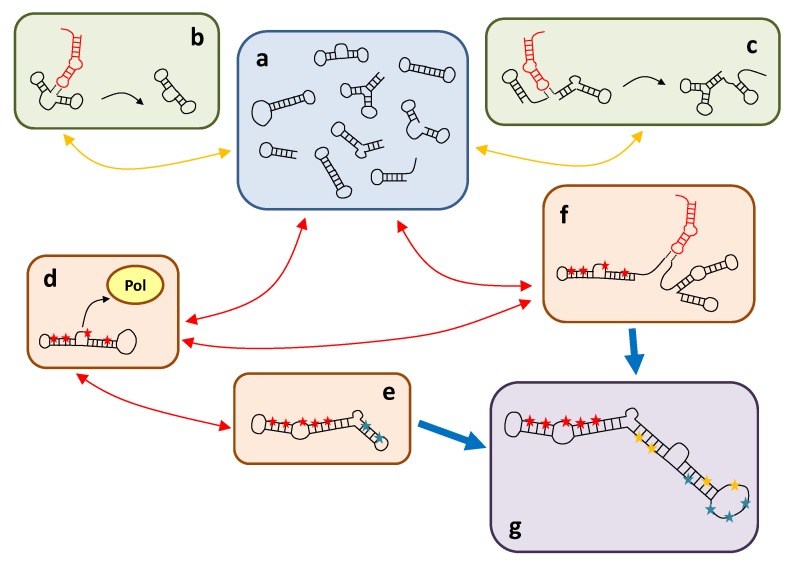
Schematics of a parsimonious scenario leading to the emergence of viroid-like replicons de novo. Structures in black represent circulating RNAs with various structures and functions; structures in red stand for hairpins with RNA ligase activity; stars indicate positions in the sequence that are fixed, different colors corresponding to possible motifs that interact with different molecules. (**a**) Circulating pool. There is a pool of RNA sequences of various origins. Short random sequences spontaneously fold into hairpin structures. (**b**) Circularization. Hairpins are able to catalyze ligation reactions, so a fraction of open chains would close in the presence of hairpins. (**c**) Modular growth. Independent RNAs can ligate through reactions analogous to those causing circularization. Both reactions in (**b**,**c**) yield novel molecules that add to the circulating pool. (**d**) A non-negligible fraction of circulating RNAs might have specific sequence positions that promote interactions with a polymerase. Once this process starts, selection for improved replication is triggered. Those RNAs would become more prevalent in the circulating pool. (**e**) Minimal circular replicons might grow in length through the random addition of nucleotides to their sequences. Sequence motifs with no specific function can evolve to improve the replicative ability of the molecule (e.g., by increasing mobility or selecting additional positions to interact with the polymerase). (**f**) New functions can be acquired through recombination of functional RNAs in the circulating pool. (**g**) Sufficiently long replicons that may arise from processes as those in (**e**) and/or (**f**) might respond to a variety of selection pressures. In practice, these replicons occupy a niche in the molecular ecology equivalent to that of viroids.

The quantitative results reported in this work give support to a de novo emergence of viroid-like replicons. We have envisaged a parsimonious scenario, summarized in Figure 4, where short RNA molecules of various origins could circularize and act as seeds of the process. There is no particular requirement in the initial condition: plant and animal cells, in particular, hold a large and variable pool of RNAs fulfilling different functions, of a variety of lengths and origins, which might in practice serve as a test bed for new functions: “A truly modern RNA World exists in each cell” [26]. Circular RNAs are ubiquitous in nature [70] and pervasively expressed in higher eukaryotes [52]. The likelihood that one such RNA bears a specific sequence that could be mistakenly recognized by an RNA polymerase is high. This recognition would be further facilitated by a compact folding mimicking dsDNA [5]. Even the template-free synthesis of RNA can be possible in certain environments [71]. In a prebiotic scenario, random RNA sequences could have fulfilled these minimal conditions, such that viroid-like replicons could have easily emerged in a precellular context. However, there are also several different extant RNAs that could be involved in this particular molecular mimicry, a prominent example being miRNAs. The compact, hairpin-like structures of miRNAs and their high diversity [54] make them good candidates to trigger such a process. Regardless of its origin, an RNA molecule that can be replicated in that way would become more abundant in front of other variants, starting in this way its Darwinian evolution towards becoming a fully-fledged selfish replicator. Nonetheless, the eventual success of such an incipient replicator can be compromised if other abilities are not developed in a timely manner. First, its replication would be initially limited to the cellular environment: acquiring the ability to move to neighboring cells seems a necessary requirement. Second, de novo replicators have to persist in a molecular ecology that might prevent their fixation in a variety of ways. In particular, if the potential niche of viroid-like replicons is already occupied, it might be extremely difficult to invade the system and displace the established molecule. The notion that niche occupation limits the success of invasions attempted by ecologically-analogous species is widespread in ecology [72,73] and should be applicable, mutatis mutandis, to molecular ecologies.

Some of the steps in the scenario above are amenable to experimental test. For example, ensembles of random RNA sequences of short length, which preferentially fold into hairpin-like structures, could be prepared and left to free interaction. At some point, the fraction of circularized molecules could be obtained by eliminating open sequences through the action of exoribonucleases. The ability of the remaining population to replicate could be assayed in an in vitro preparation containing an RNA replicase and free nucleotides.

Subsequent evolution of short replicating RNAs could have occurred through several different, not mutually exclusive, mechanisms. One is elongation through the addition of stretches of nucleotides. In Spiegelman’s evolutionary experiment, where the unique selective pressure applied favored faster replication, the length of RNA replicators decreased through evolution [25]. However, this reduction does not need to occur in more complex environments, where other selective pressures might be acting. In some of our in silico evolution experiments, we monitored the appearance of multiple sequence motifs. In a natural environment, it cannot be discarded that initiating replication at more than one site confers an advantage that compensates for the mutational cost of replicating longer molecules. Furthermore, longer sequences can respond to a high number of selection pressures, thus paving the way for the emergence of specific sequence motifs able to fulfill new functions: interaction with other molecules, cell-to-cell movement or improved replication through additional structural motifs are a few examples. Similarly, the duplication of parts of the sequence through imperfect rolling circle replication can be sources of novelty through neo- or sub-functionalization, as happens with gene duplication. Examples of such processes of genome length increase have been previously documented in the coleviroids [74], the genesis of CCCVd variants containing duplicated segments of the left terminal domain and part of the adjacent variable domain [75], and the long CEVd isolate D-104 [76].

Modular evolution appears as a plausible and highly-efficient mechanism to acquire new functions. Repeated recombination between functional modules selected in different contexts [66] could have facilitated the transmission of ubiquitous functions such as cleavage through hammerheads, ligation catalysis through hairpins, or long-distance movement motifs. In this context, one wonders whether the similarity in sequence and structure of different viroids and viroid-related replicons has to be interpreted as a result of descent with modification or of horizontal transfer. In the latter case, functional submolecular elements could be better described as the nodes of a network that underlies and favors the rapid emergence of new viroid-like replicons through shuffling of functional modules that are quite abundant in the molecular ecosystem [67,68,69]. This idea has sound support in viruses [77] and may underlie the evolutionary emergence of multipartite viral genomes [78]. Examples of such recombinant origin in viroids are best illustrated by CLVd [63], which resulted from the intracellular recombination between a *Hostuviroid* and a *Pospiviroid* coinfecting the same plant, and AGVd, which resulted from recombination of GYSVd-1 (an *Apscaviroid*) and CEVd (a *Pospiviroid*), both infecting grapevine plants [64].

The emergence of genetic parasites is unavoidable [79,80]. In the RNA World, primitive selfish replicons must have emerged, and they might well have been viroid-like. However, the ease with which this kind of parasite seems to arise suggests that this replicative strategy might have been discovered multiple times in evolution. Retroviroid-like elements provide indirect support to this hypothesis, since they should have appeared following the evolutionary discovery of DNA [5]. Though it has been commonly accepted that Pospiviroidae and Avsunviroidae had a common phylogenetic origin after [32], the cell nucleus and chloroplasts offer significantly different environments wherein viroid-like parasites could proliferate [81]. Therefore and though our results cannot override a possible monophyletic origin, an independent origin of the two viroid families can neither be discarded. In the context of an RNA World, chemistry had a completely different nature, DNA was absent, and all current proteins did not exist as such. Extant viroids may resemble in many ways early replicons in an RNA World, but this similarity does not imply that the former are linked by descent to the latter (see E.V. Koonin’s comment in [9]). The intimate relationship between viroid sequences and the extant molecules they interact with, together with their fast adaptive responses to new selection pressures, speaks to the unlikelihood of maintaining specific sequences unaltered for billions of years. In the absence of null models able to quantify the extent of conservation of active sites coevolving with other cellular molecules and the degree of convergent sequence evolution in macroevolutionary times, the support in favor of an old or a new origin remains circumstantial. Still, computational approaches as those used in this work are able to quantify the likelihood of specific steps involved in a de novo emergence of viroid-like replicons, supporting the plausibility of an evolutionary pathway that is not only likely, but also simple and consistent at once.

## Figures and Tables

**Table 1:** Structural properties of viroids of length *ℓ*. We list their G + C content, the minimal folding energy *E* in kcal mol${}^{-1}$ (see Materials and Methods), and the number *h* of hairpins and *p* of base pairs. For comparison, the expected values of the number of hairpins, E(h), and base pairs, E(p), in exact calculations of structures of each length (cf. Equation (6)) are reported along the actual values.

Family	Genus	Species	*ℓ*	G + C %	E	*h*	E(h)	*p*	E(p)
Avsunviroidae	*Avsunviroid*	ASBVd	247	38.1	−88.8	3	10.0	84	71.5
	*Elaviroid*	ELVd	335	54.0	−179.0	5	13.4	118	96.7
	*Pelamoviroid*	CChMVd	399	55.4	−204.2	12	15.8	140	115.1
		PLMVd	337	52.5	−185.8	8	13.4	121	97.3
Pospiviroidae	*Apscaviroid*	ADFVd	306	58.5	−157.1	2	12.3	111	88.4
		ASSVd	329	60.5	−152.8	2	13.1	110	95.0
		AGVd	369	58.0	−199.2	2	14.7	135	106.5
		CBLVd	318	59.4	−151.3	2	12.7	106	91.9
		CDVd	294	54.4	−136.8	2	11.8	101	85.0
		CVd-V	294	60.2	−146.9	2	11.8	102	85.0
		CVd-VI	330	60.0	−163.3	3	13.2	109	95.3
		GYSVd	367	61.0	−177.7	2	14.6	132	105.9
		GYSVd-1	363	60.0	−171.6	7	14.4	133	104.8
		GYSVd-2	361	59.6	−169.6	7	14.4	132	104.2
		PBCVd	315	60.6	−148.1	5	12.6	109	91.0
		PVd-2	358	59.8	−176.7	6	14.2	128	103.3
	*Cocadviroid*	CBCVd	284	55.6	−145.7	2	11.4	102	82.1
		CCCVd	246	59.3	−131.6	2	10.0	84	71.2
		CTiVd	254	60.2	−125.8	2	10.3	81	73.5
		HLVd	256	57.0	−113.6	2	10.4	85	74.1
	*Coleviroid*	CBVd	295	52.9	−141.3	2	11.9	107	85.3
		CBVd-1	248	55.2	−105.7	2	10.1	90	71.8
		CBVd-2	301	60.1	−161.4	4	12.1	114	87.0
		CBVd-3	361	55.7	−194.7	2	14.4	134	104.2
	*Hostuviroid*	DLVd	342	58.8	−172.4	2	13.6	120	98.7
		HSVd	297	56.2	−128.4	2	11.9	101	85.9
	*Pospiviroid*	CSVd	354	52.8	−181.5	2	14.1	125	102.2
		CEVd	371	60.4	−204.9	2	14.7	135	107.1
		CLVd	370	58.1	−188.9	2	14.7	128	106.8
		IrVd	370	61.6	−198.4	2	14.7	131	106.8
		MPVd	360	58.9	−189.0	2	14.3	126	103.9
		PCFVd	348	59.2	−189.4	2	13.9	125	100.5
		PSTVd	359	58.2	−188.5	2	14.3	129	103.6
		TASVd	360	55.6	−188.3	2	14.3	133	103.9
		TCDVd	360	56.7	−181.4	2	14.3	127	103.9
		TPMVd	360	57.5	−182.7	2	14.3	123	103.9
Unclassified		AFCVd	372	56.7	−185.9	2	14.8	132	107.3
		CVd-VII	368	52.2	−173.0	3	14.6	128	106.2
		GLVd	328	58.2	−154.4	4	13.1	109	94.7
		PVd	396	57.8	−201.7	5	15.7	143	114.2
		RVd-I	361	58.4	−191.8	2	14.4	126	104.2

**Table 2:** Number of circular RNA structures of length *ℓ*, exhibiting *h* hairpins and *p* base pairs.

*ℓ*	h	p=2	*3*	*4*	*5*	*6*	*7*	*8*	*9*	*10*	*11*
10	*2*	1									
11	*2*	1									
12	*2*	2	1								
13	*2*	2	1								
14	*2*	3	2	1							
15	*2*	3	2	2							
16	*2*	4	3	6	1						
17	*2*	4	3	10	3						
18	*2*	5	4	19	9	1					
19	*2*	5	4	28	18	4					
20	*2*	6	5	44	33	16	1				
21	*2*	6	5	60	53	36	5				
	*3*					1					
22	*2*	7	6	85	81	82	23	1			
	*3*					2					
23	*2*	7	6	110	116	151	64	6			
	*3*					7	1				
24	*2*	8	7	146	161	276	157	34	1		
	*3*					20	6				
25	*2*	8	7	182	215	452	322	106	7		
	*3*					42	21	2			
26	*2*	9	8	231	281	731	614	294	45	1	
	*3*					84	56	14			
27	*2*	9	8	280	358	1106	1068	665	166	8	
	*3*					156	126	57	4		
28	*2*	10	9	344	449	1652	1773	1401	507	60	1
	*3*					264	252	176	28		
	*4*							1			
29	*2*	10	9	408	553	2360	2783	2668	1279	248	9
	*3*					429	462	456	130	5	
	*4*							2			

## Abbreviations

The following abbreviations are used in this manuscript. We list as well the NCBI accession numbers of viroid sequences:

ADFVd	*Apple dimple fruit viroid*	X99487.1	
AFCVd	*Apple fruit crinkle viroid*	AB429213.1	
AGVd	*Australian grapevine viroid*	X17101.1	
ASBVd	*Avocado sunblotch viroid*	X13000.1	
ASSVd	*Apple scar skin viroid*	X17696.1	
CBVd	*Coleus blumei viroid*	X97202.1	
		X52960.1	(CBVd-1)
		X95365.1	(CBVd-2)
		X95364.1	(CBVd-3)
CBCVd	*Citrus bark cracking viroid*	MG457794.1	
CBLVd	*Citrus bent leaf viroid*	M74065.1	
CVd	*Citrus viroid*	EF617306.1	(CVd-V)
		AB019508.1	(CVd-VI)
		KX013553.1	(CVd-VII)
CCCVd	*Coconut cadang-cadang viroid*	J02049.1	
CChMVd	*Chrysanthemum chlorotic mottle viroid*	Y14700.1	
CDVd	*Citrus dwarfing viroid*	AF184147.1	
CEVd	*Citrus exocortis viroid*	M34917.1	
CLVd	*Columnea latent viroid*	X15663.1	
CSVd	*Chrysanthemum stunt viroid*	AJ001849.1	
CTiVd	*Coconut trinangaja viroid*	M20731.1	
DLVd	*Dahlia latent viroid*	MG214159.1	
ELVd	*Eggplant latent viroid*	AJ536612.1	
GLVd	*Grapevine latent viroid*	KR605505.1	
GYSVd	*Grapevine yellow speckle viroid*	X06904.1	
		J04348.1	(GYSVd-1)
		KF916050.1	(GYSVd-2)
HLVd	*Hop latent viroid*	X07397.1	
HSVd	*Hop stunt viroid*	X00009.1	
IrVd	*Iresine viroid*	X95734.1	
MPVd	*Mexican papita viroid*	L78454.1	
PVd	*Persimmon viroid*	AB366022.1	
		AB817729.1	(PVd2)
PBCVd	*Pear blister canker viroid*	D12823.1	
PCFVd	*Pepper chat fruit viroid*	FJ409044.1	
PLMVd	*Peach latent mosaic viroid*	M83545.1	
PSTVd	*Potato spindle tuber viroid*	V01465.1	
RVd	*Rubber viroid*	HM107844.1	(RVd-I)
TASVd	*Tomato apical stunt viroid*	K00818.1	
TCDVd	*Tomato chlorotic dwarf viroid*	AF162131.1	
TPMVd	*Tomato planta macho viroid*	K00817.1	
